# s-Afadin binds to MAGUIN/Cnksr2 and regulates the localization of the AMPA receptor and glutamatergic synaptic response in hippocampal neurons

**DOI:** 10.1016/j.jbc.2023.103040

**Published:** 2023-02-18

**Authors:** Tomohiko Maruo, Kiyohito Mizutani, Muneaki Miyata, Toshihiko Kuriu, Shotaro Sakakibara, Hatena Takahashi, Daichi Kida, Kouki Maesaka, Tsukiko Sugaya, Ayuko Sakane, Takuya Sasaki, Yoshimi Takai, Kenji Mandai

**Affiliations:** 1 Department of Molecular and Cellular Neurobiology, Kitasato University Graduate School of Medical Sciences, Sagamihara, Kanagawa, Japan; 2 Department of Biochemistry, Kitasato University School of Medicine, Sagamihara, Kanagawa, Japan; 3 Division of Pathogenetic Signaling, Department of Biochemistry and Molecular Biology, Kobe University Graduate School of Medicine, Kobe, Hyogo, Japan; 4 Department of Biochemistry, Tokushima University Graduate School of Medical Sciences, Tokushima, Japan; 5 Research and Development Center, Osaka Medical and Pharmaceutical University, Takatsuki, Osaka, Japan; 6 Department of Interdisciplinary Researches for Medicine and Photonics, Institute of Post-LED Photonics, Tokushima University, Tokushima, Japan

**Keywords:** scaffold protein, synapse, electrophysiology, AMPA receptor, epilepsy

## Abstract

A hippocampal mossy fiber synapse implicated in learning and memory is a complex structure in which a presynaptic bouton attaches to the dendritic trunk by puncta adherentia junctions (PAJs) and wraps multiply branched spines. The postsynaptic densities (PSDs) are localized at the heads of each of these spines and faces to the presynaptic active zones. We previously showed that the scaffolding protein afadin regulates the formation of the PAJs, PSDs, and active zones in the mossy fiber synapse. Afadin has two splice variants: l-afadin and s-afadin. l-Afadin, but not s-afadin, regulates the formation of the PAJs but the roles of s-afadin in synaptogenesis remain unknown. We found here that s-afadin more preferentially bound to MAGUIN (a product of the *Cnksr2* gene) than l-afadin *in vivo* and *in vitro*. *MAGUIN/CNKSR2* is one of the causative genes for nonsyndromic X-linked intellectual disability accompanied by epilepsy and aphasia. Genetic ablation of *MAGUIN* impaired PSD-95 localization and α-amino-3-hydroxy-5-methyl-4-isoxazolepropionic (AMPA) receptor surface accumulation in cultured hippocampal neurons. Our electrophysiological analysis revealed that the postsynaptic response to glutamate, but not its release from the presynapse, was impaired in the *MAGUIN*-deficient cultured hippocampal neurons. Furthermore, disruption of *MAGUIN* did not increase the seizure susceptibility to flurothyl, a GABA_A_ receptor antagonist. These results indicate that s-afadin binds to MAGUIN and regulates the PSD-95-dependent cell surface localization of the AMPA receptor and glutamatergic synaptic responses in the hippocampal neurons and that MAGUIN is not involved in the induction of epileptic seizure by flurothyl in our mouse model.

Synapses are structures through which neurons transmit impulses to other neurons or cells. A synapse is composed of multiple cell adhesion sites, including synaptic junctions (SJs) and puncta adherentia junctions (PAJs) that are similar to epithelial adherens junctions. A prominent example of a synapse is the hippocampal mossy fiber synapse, a large and complex structure formed between the axon terminal of dentate granule cell and the proximal dendrite of CA3 pyramidal cell (1, 2). At the mossy fiber synapse, presynaptic boutons attach to a dendritic shaft by multiple PAJs and wrap around a highly branched dendritic spine, where multiple SJs are formed (3). The postsynaptic densities (PSDs) are located at the heads of the spine branches and face toward the active zones (AZs). In mice, there are approximately twenty AZs in a single mossy fiber bouton.

Afadin (a product of the *Afdn* gene) was originally purified from rat brains as an actin filament (F-actin)-binding protein and displayed a primary sequence similar to that of the human *ALL-1 fused gene from chromosome 6* (*AF-6*) gene product (4). It was purified as the larger and smaller variants with molecular masses of 205 kDa and 190 kDa, named l-afadin and s-afadin, respectively. From the N-terminus, l-afadin has multiple domains, including two Ras-associating (RA) domains, a forkhead-associated domain, a dilute domain, a PDZ domain, three proline-rich regions, and an F-actin-binding (FAB) domain, whereas s-afadin lacks the C-terminal FAB domain and the third proline-rich region (5). l-Afadin is broadly expressed in all tissues examined, whereas s-afadin is only expressed in the brain (4).

Both l-afadin and s-afadin bind to cell adhesion molecule nectin, which constitutes a family of four members (nectin-1, nectin-2, nectin-3, and nectin-4) (5). In the hippocampal mossy fiber synapse PAJs, l-afadin as well as N-cadherin and αN-catenin is symmetrically localized at the presynaptic and postsynaptic sides, whereas nectin-1 and nectin-3 are asymmetrically localized at the presynaptic and postsynaptic sides, respectively (6, 7). In addition to this localization, l-afadin and presumably s-afadin are localized partly at the SJs in the dendritic spines (8, 9, 10). Our recent studies on the *in vivo* and *in vitro* roles of afadin in the structural and functional differentiations of the hippocampal synapses showed that the localization of nectin-1, nectin-3, and N-cadherin is impaired in the *afadin*-deficient synapses. Further, the studies by serial block face-scanning electron microscopy showed that the complexity of postsynaptic spines and mossy fiber boutons, the number of spine heads, the area of the PSDs, and the density of the synaptic vesicles docked to the AZs are decreased in the *afadin*-deficient synapses (11). These results indicate that afadin plays multiple roles in the complex ultrastructural morphogenesis of the hippocampal mossy fiber synapses. Consistent with these morphological results, the electrophysiological studies revealed that both the release probability of glutamate and the postsynaptic responsiveness to glutamate are significantly reduced in the *afadin*-deficient mossy fiber synapses and in the synapses in cultured *afadin*-deficient hippocampal neurons (12). These results indicate that afadin plays multiple roles in the presynaptic and postsynaptic functions of the hippocampal synapses. However, it remains unknown which splice variant of afadin, l-afadin or s-afadin, is involved in the structural and functional differentiations of the mossy fiber synapses.

Using the *afadin*-deficient cultured hippocampal neurons in which the PAJ-like structure was poorly formed, we subsequently showed that l-afadin, but not s-afadin, is involved in the formation of the PAJs through the binding to F-actin and α-catenin (13). Thus, the roles of s-afadin in the structural and functional differentiations of the hippocampal synapses including the mossy fiber synapses remain unknown.

Extending these earlier findings, we attempted here to elucidate the roles of s-afadin in the formation of the hippocampal synapses and found that s-afadin more preferentially bound to MAGUIN than l-afadin *in vivo* and *in vitro* and regulated the structural and functional differentiations of the PSDs through the binding to MAGUIN. MAGUIN was originally identified as an S-SCAM-binding protein (14). There are two splice variants for MAGUIN; a longer isoform MAGUIN-1 and a shorter isoform MAGUIN-2 that lacks the C-terminal 137 amino acids (aa) of MAGUIN-1. MAGUIN is a mammalian homologue of the *Drosophila Cnk* gene product, which regulates the Ras-Raf–MAPK signaling pathway (15). MAGUIN was further shown to bind to Raf-1 and PSD-95 (14, 16). Clinically, the partial deletion or deficiency of *MAGUIN/CNKSR2* can be a cause of nonsyndromic X-linked intellectual disability and seizure (17, 18, 19, 20). We also found here that s-afadin regulated the clustering of PSD-95 and cell surface localization of the α-amino-3-hydroxy-5-methyl-4-isoxazolepropionic (AMPA) receptor, that MAGUIN regulated glutamatergic synaptic responses in the hippocampal neurons and that disruption of *MAGUIN* did not increase the seizure susceptibility to flurothyl in our mouse model.

## Results

### Identification of MAGUIN as an s-afadin-binding protein

**Figure 1:**
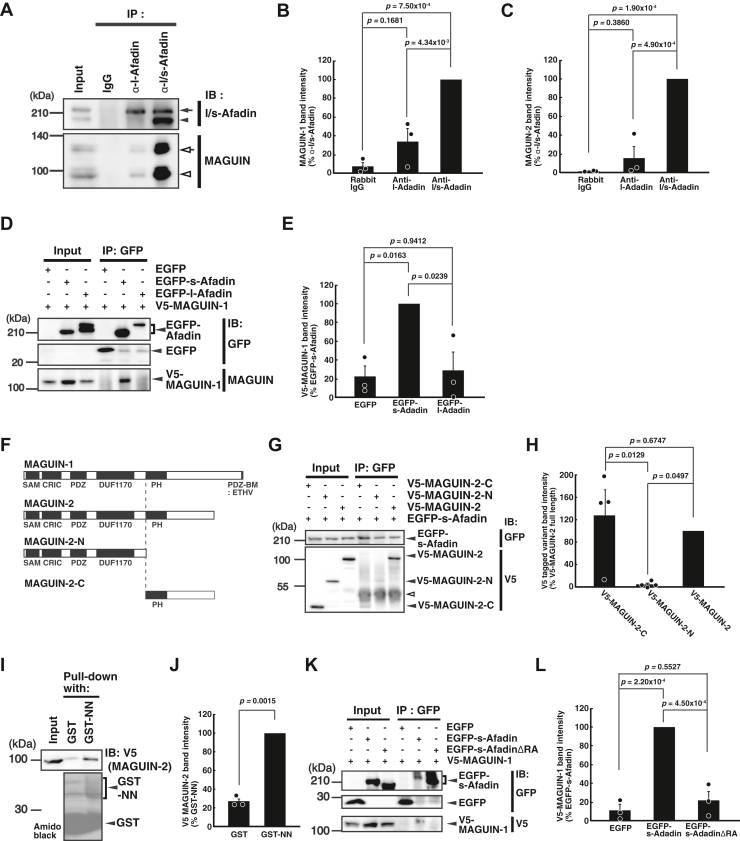
**Binding of MAGUIN to s-afadin.***A*–*E*, preferential binding of MAGUIN to s-afadin over l-afadin. *A*–*C*, *in vivo* immunoprecipitation assay for the binding of MAGUIN to s-afadin. The lysate of the light membrane fraction of the mouse brains was immunoprecipitated by rabbit IgG, the l-afadin pAb, or the l/s-afadin pAb. The lysate and the immunoprecipitated samples were subjected to SDS-PAGE, followed by Western blotting with the l/s-afadin pAb and the MAGUIN pAb recognizing both of MAGUIN-1 and MAGUIN-2. *A*, Western blot. *Arrow*, l-afadin; *arrowhead*, s-afadin; *open arrow*, MAGUIN-1; *open arrowhead*, MAGUIN-2. *B* and *C*, quantification of band intensities in three independent experiments. *B*, the average ratio of MAGUIN-1 immunoprecipitated with each indicated Ab to MAGUIN-1 immunoprecipitated with the l/s-afadin pAb. *p* = 0.0006 (one way ANOVA). *C*, the average ratio of MAGUIN-2 immunoprecipitated with each indicated Ab to MAGUIN-2 immunoprecipitated with the l/s-afadin pAb. *p* = 0.0001 (one way ANOVA). *D* and *E*, *in vitro* immunoprecipitation assay for the binding of MAGUIN to s-afadin. The lysates from HEK293 cells expressing EGFP, EGFP-tagged s-afadin, or EGFP-tagged l-afadin with V5-tagged MAGUIN-1 were immunoprecipitated with the GFP pAb. The cell lysates and immunoprecipitated samples were subjected to Western blotting with the GFP pAb and the MAGUIN pAb. *D*, Western blot. *E*, quantification of band intensities in three independent experiments. The average ratio of V5-tagged MAGUIN-1 immunoprecipitated with each indicated recombinant protein to V5-tagged MAGUIN-1 immunoprecipitated with EGFP-s-afadin. *p* = 0.0101 (one way ANOVA). *F*–*H*, binding of the C-terminal portion of MAGUIN-2 to s-afadin. *F*, schematic drawings for the full length and deletion mutants of MAGUIN used in the experiments. SAM, the sterile alpha motif domain; CRIC, the conserved region in CNK domain; PDZ, the PDZ domain; DUF1170, the domain of unknown function 1170; PH, the pleckstrin homology domain; PDZ-BM, the PDZ binding motif. Note that MAGUIN-2 lacks the C-terminal 137 aa of MAGUIN-1 including the PDZ binding motif (ETHV). *G* and *H*, *in vitro* immunoprecipitation assay for the binding of the C-terminal portion of MAGUIN-2 to s-afadin. The lysates from HEK293 cells expressing V5-tagged MAGUIN-2, V5-tagged MAGUIN-2-C, or V5-tagged MAGUIN-2-N with EGFP-tagged s-afadin were immunoprecipitated with the GFP pAb. The cell lysates and immunoprecipitated samples were subjected to Western blotting with the GFP pAb and the V5 mAb. *G*, Western blot. *Open arrowhead*, nonspecific signals. *H*, quantification of band intensities in four independent experiments. The average ratio of each V5-tagged MAGUIN-2 variant immunoprecipitated with EGFP-s-afadin to V5-tagged MAGUIN-2 immunoprecipitated with EGFP-s-afadin. *p* = 0.0100 (one-way ANOVA). *I*–*L*, binding of MAGUIN to the N-terminal RA domains of afadin. *I* and *J*, GST pull-down assay. The lysate of HEK293 cells expressing V5-tagged MAGUIN-2 was subjected to GST pull-down assay using GST or GST-NN (the RA domains of afadin). The cell lysate and the samples pulled down were analyzed by Western blotting with the V5 mAb and followed by amido black staining. *I*, Western blot and amido black staining. *J*, quantification of band intensities in three independent experiments. The average ratio of V5-tagged MAGUIN-2 pulled down by GST to V5-tagged MAGUIN-2 pulled down by GST-NN. Statistical difference was determined by two-tailed paired *t* test. *K* and *L*, *in vitro* immunoprecipitation assay for the binding of MAGUIN to the RA domains of s-afadin. The lysates from HEK293 cells expressing EGFP, EGFP-tagged s-afadin, or EGFP-tagged s-afadinΔRA with V5-tagged MAGUIN-1 were immunoprecipitated with the GFP pAb. s-AfadinΔRA, s-afadin lacking the RA domains. The cell lysates and immunoprecipitated samples were subjected to Western blotting with the GFP pAb and the V5 mAb. *K*, Western blot. *L*, quantification of band intensities in three independent experiments. The average ratio of V5-tagged MAGUIN-1 immunoprecipitated with each indicated recombinant protein to V5-tagged MAGUIN-1 immunoprecipitated with EGFP-s-afadin. *p* = 0.0001 (one-way ANOVA). *IB*, immunoblotting; *IP*, immunoprecipitation; *RA*, the Ras-associating domain. Images are a representative of three independent experiments in *A*, *D*, *I*, and *K*, or four independent experiments in *G*. The *p*-values of the paired differences by post hoc Scheffe tests are shown in *B*, *C*, *E*, *H*, and *L*. Error bars, SEM. EGFP, enhanced GFP; HEK, human embryonic kidney; pAb, polyclonal antibody; PH, pleckstrin homology; RA, Ras-associating.

To elucidate the mechanism for the formation of the PSDs by l-afadin and s-afadin, we attempted to identify afadin-binding proteins using an immunoprecipitation assay with the l-afadin polyclonal antibody (pAb) and the l/s-afadin pAb recognizing both l-afadin and s-afadin in the crude membrane fraction prepared from the mouse brains. The immunoprecipitates were subjected to SDS-PAGE, followed by Western blotting. l-Afadin was immunoprecipitated with both the pAbs, whereas s-afadin was immunoprecipitated by an l/s-afadin pAb but not an l-afadin pAb (Fig. 1*A*). We found that MAGUIN-1 and MAGUIN-2 were coimmunoprecipitated with afadin. The amounts of MAGUIN-1 and MAGUIN-2 immunoprecipitated by the l/s-afadin pAb were higher than those immunoprecipitated by the l-afadin pAb (Fig. 1, *A*−*C*). This more preferential binding of MAGUIN to s-afadin than to l-afadin was confirmed by immunoprecipitation assays in which each of enhanced GFP (EGFP)-tagged afadin splice variants and V5-tagged MAGUIN-1 were coexpressed in human embryonic kidney (HEK) 293 cells (Fig. 1, *D* and *E*). V5-tagged MAGUIN-1 was immunoprecipitated with EGFP-tagged s-afadin, while V5-tagged MAGUIN-1 was hardly immunoprecipitated with EGFP-tagged l-afadin. These results indicate that s-afadin binds to MAGUIN-1 more preferentially than l-afadin *in vivo* and *in vitro*.

### Binding regions of s-afadin and MAGUIN and requirement of the binding of s-afadin to MAGUIN for the localization of PSD-95 in cultured hippocampal neurons

Because MAGUIN-1 is known to interact with the PSD components S-SCAM and PSD-95 (14), we examined whether the binding of s-afadin to MAGUIN-1 is required for the formation of the PSDs in cultured hippocampal neurons. For this purpose, we first determined the s-afadin-binding region of MAGUIN. The V5-tagged full-length and N-terminal and C-terminal fragments of MAGUIN-2, a shorter splice variant of MAGUIN, shown in Figure 1*F* were coexpressed with EGFP-s-afadin in HEK293 cells and EGFP-s-afadin was immunoprecipitated from these cell lysates with a GFP pAb. MAGUIN-2 was used because the PDZ-binding motif in MAGUIN-1 was not essential for the binding of afadin to MAGUIN-1/-2 (Fig. 1, *A* and *C*). The C-terminal fragment of MAGUIN-2 containing the pleckstrin homology (PH) domain was coimmunoprecipitated with EGFP-s-afadin but not with the N-terminal fragment (Fig. 1, *G* and *H*), indicating that s-afadin binds to the C-terminal region of MAGUIN-2, which contains a PH domain. Next, we determined the MAGUIN-binding region of s-afadin. Because the PH domain of another afadin-binding protein, PLEKHA7, binds to the RA domains of afadin (21), we further examined the possibility of the binding of the RA domains of afadin to MAGUIN-2. The cell lysate obtained from HEK293 cells expressing V5-tagged MAGUIN-2 was subjected to a pull-down assay using glutathione S-transferase (GST)-afadin-NN fragment (aa 2–500) containing the RA domains of afadin. MAGUIN-2 bound to the GST-afadin-NN fragment (Fig. 1, *I* and *J*). Consistently, the mutant of s-afadin lacking the RA domains was not coimmunoprecipitated with GFP-MAGUIN-1 (Fig. 1, *K* and *L*). Collectively, these results indicate that the binding of s-afadin to MAGUIN is mediated through the first and/or second RA domain(s) of s-afadin and the C-terminal region of MAGUIN (aa 567–897) containing the PH domain.

**Figure 2:**
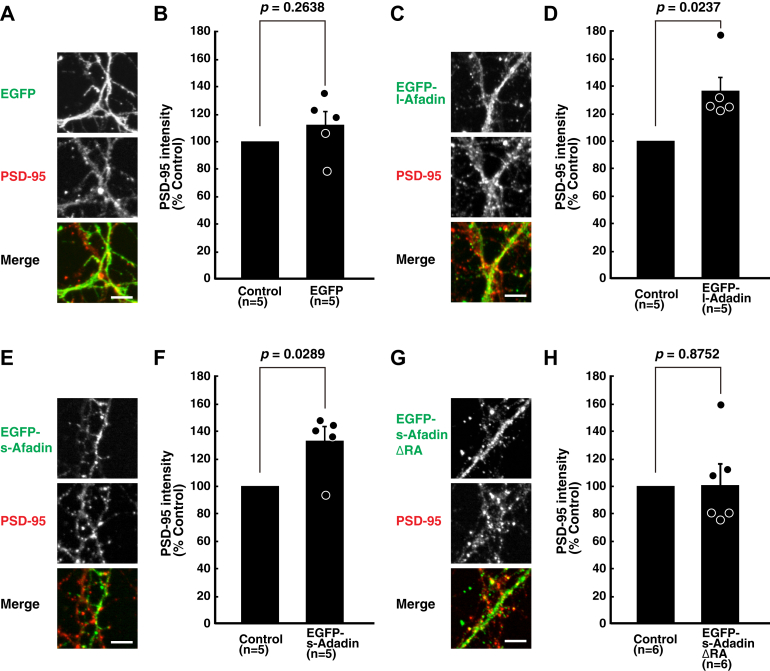
**Requirement of the RA domains of s-afadin for the localization of PSD-95 incultured hippocampal neurons.***A*, *C*, *E*, and *G*, restoration of the accumulation of PSD-95 by expression of l-afadin or s-afadin but not by s-afadin lacking RA domains. Cultured hippocampal neurons were stained with the PSD-95 mAb and GFP pAb. The neurons were obtained from the *afadin*-deficient embryos (*Afdn*^f/f^;*nestin*-cre) at embryonic day 18.5 and were transfected with the constructs to express the indicated recombinant proteins. The scale bars represent 5 μm. *B*, *D*, *F*, and *H*, the average ratio of the intensity of signals for PSD-95 in the neurons transfected with the constructs expressing the indicated recombinant proteins, relative to that of the adjacent untransfected neurons in the *afadin*-deficient hippocampal neuron culture. Each data point represents the mean signal intensity of 15 PSD-95 clusters. Five or six neuron pairs were measured to examine the effect of reexpressions of each EGFP-afadin variant in *afadin*-deficient neurons in primary cultures prepared twice. *RA*, the Ras-associating domain. Statistical difference was determined against EGFP transfected control neurons by two-tailed paired *t* test. Error bars, SEM. EGFP, enhanced GFP; pAb, polyclonal antibody; PSD, postsynaptic density.

We then examined whether reexpression of l-afadin, s-afadin, or the s-afadin mutant lacking the RA domains restore the formation of the PSD-like structures in the cultured *afadin*-deficient hippocampal neurons obtained from *Afdn*^f/f^;*nestin*-cre embryos. Reexpression of l-afadin or s-afadin significantly increased the signals for PSD-95, compared with those of the adjacent untransfected neurons (Fig. 2, *A*−*F*). In contrast, reexpression of the s-afadin mutant lacking the RA domains did not restore the immunofluorescence signals for PSD-95 at synapses, compared with those of the adjacent untransfected neurons (Fig. 2, *G* and *H*). These results indicate that the binding of MAGUIN-1 to s-afadin regulates the localization of PSD-95 in cultured hippocampal neurons.

### Requirement of MAGUIN-1 for the localization of PSD-95 in cultured hippocampal neurons

**Figure 3:**
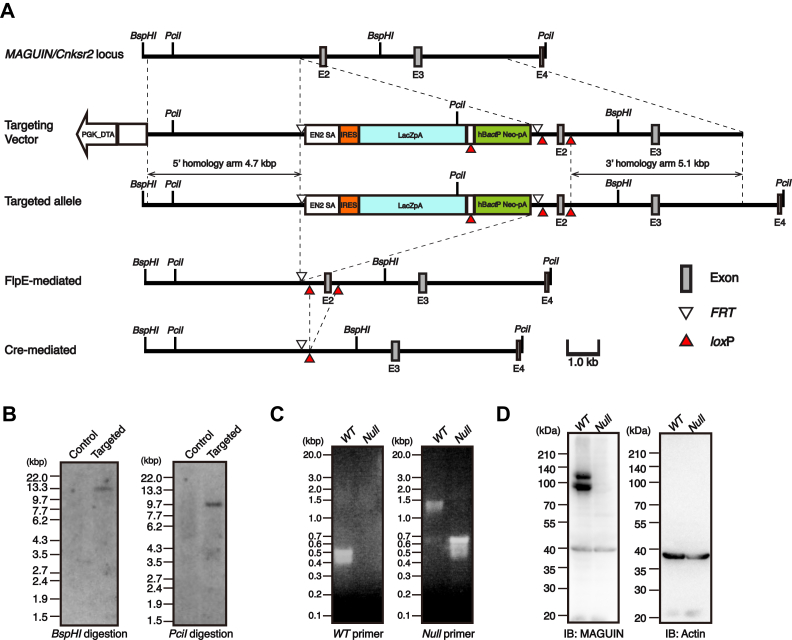
**Generation of a *MAGUIN/Cnksr2* mutant allele.***A*, partial structure of the mouse *MAGUIN/Cnksr2* locus and region targeted for gene disruption. Schematics are shown for the *WT* genomic locus, targeting vector, targeted allele, targeted allele after FlpE-mediated recombination, and targeted allele after FlpE-mediated recombination followed by cre-mediated recombination. The mice generated from correctly targeted C57BL/6N-derived ES cells were crossed with mice expressing FlpE recombinase in the germ cell lineage to excise the LacZ-Neo cassette. The mutant mice obtained through this cross were then crossed with mice expressing cre recombinase in the germ cell lineage to excise the *lox*P-flanked exon 2. Finally, the mice with a frameshift mutation in the *MAGUIN/Cnksr2* gene locus were obtained. *B*, Southern blot analysis of the targeted ES clone. *BspHI*- or *Pcil*-digested genomic DNA derived from ES cells were hybridized with a *Neo* probe. Control DNA was obtained from the parental ES cells. For each blot, images of the DNA ladder marker lane and Southern blot were separately taken and reconstructed to show the molecular weight of the DNA. *C*, PCR genotyping of *MAGUIN*-deficient mice. *D*, Western blot analysis of the brain homogenates prepared from the control *WT* and *MAGUIN*-deficient mice with the MAGUIN pAb. The actin mAb was used as a loading control. *DTA*, diphtheria toxin A; E, exon; *EN2 SA*, *Engrailed-2* splice acceptor; *FRT*, flippase recognition target; *hBactP*, human β-actin promoter; *IB*, immunoblotting; *IRES*, internal ribosomal entry site; *Neo*, aminoglycoside 3′-phosphotransferase (neomycin resistance) gene; *Null*, *MAGUIN*-deficient; *pA*, polyadenylation signal; pAb, polyclonal antibody; *PGK*, phosphoglycerate kinase 1 promoter.

To determine whether MAGUIN is required for the localization of PSD-95 in cultured neurons, we generated a *MAGUIN* null allele by homologous recombination (Fig. 3, *A*–*C*). The *MAGUIN* gene locus is on the X chromosome in the mouse. The male and female *MAGUIN*-deficient mice were born in the expected Mendelian ratios, grew up into adult, and were fertile. The general appearance of the male hemizygous and female heterozygous and homozygous mice was indistinguishable from those of the control *WT* mice. In Western blot analysis of the brain homogenate prepared from the *MAGUIN*-deficient mouse, no bands corresponding to MAGUIN were detected (Fig. 3*D*).

**Figure 4:**
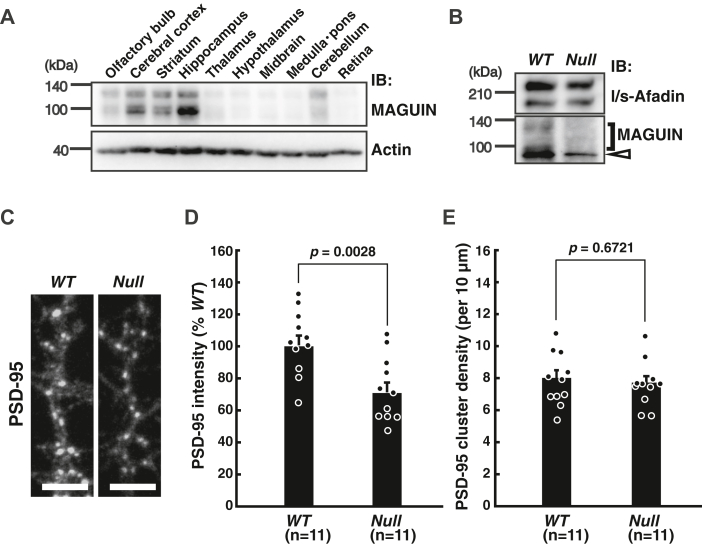
**Requirement of MAGUIN for the localization of PSD-95 incultured hippocampal neurons.***A*, expression of MAGUIN in the various mouse brain regions. Lysates of each dissected region of the mouse brain were subjected to SDS-PAGE, followed by Western blotting with the MAGUIN pAb and actin mAb for a loading control. *B*, expression of MAGUIN in mouse hippocampal neuron cultures. Lysates of the hippocampal cultures of the indicated genotypes were subjected to SDS-PAGE, followed by Western blotting with the l/s-afadin pAb and MAGUIN pAb. *Open arrowhead*, nonspecific signals observed in the cultures. *C*–*E*, reduced signal intensity of the PSD-95 clusters and no reduction in the density of the PSD-95 clusters in the *MAGUIN*-deficient hippocampal neurons. *C*, the cultured hippocampal neurons stained with the PSD-95 mAb. The scale bars represent 5 μm. *D*, reduction of the signal intensity of the PSD-95 clusters in the *MAGUIN*-deficient neurons. Each data point represents the mean signal intensity of 50 PSD-95 clusters per neuron. Eleven control and 11 *MAGUIN*-deficient neurons were measured in the primary hippocampal neuron cultures prepared three times. *E*, no reduction in the PSD-95 cluster density in the *MAGUIN*-deficient neurons. Each data point represents the mean density of PSD-95 clusters along dendrites per 10 μm per neuron. Eleven control and 11 *MAGUIN*-deficient neurons were measured in the primary hippocampal neuron cultures prepared three times. *WT* littermates were used as controls in *B*–*E*. *IB*, immunoblotting; *Null*, *MAGUIN*-deficient. Statistical difference was determined by two-tailed Student’s *t* test. Error bars, SEM. pAb, polyclonal antibody; PSD, postsynaptic density.

Because a MAGUIN Ab applicable to immunostaining is not available, we determined whether MAGUIN is expressed in the hippocampus *in vivo*. For this purpose, the expression of MAGUIN in the various mouse brain regions was examined by Western blotting. MAGUIN was found to be most highly expressed in the hippocampus, next highly expressed in the striatum and cerebral cortex, and moderately expressed in the olfactory bulb and cerebellum (Fig. 4*A*).

To investigate the role of MAGUIN in the localization of PSD-95 at synapses *in vitro*, hippocampal neuron cultures were prepared from control *WT* embryos and *MAGUIN*-deficient embryos. Afadin expression was not affected by the deletion of *MAGUIN* in the hippocampal neuron culture prepared from the *MAGUIN*-deficient embryos (Fig. 4*B*). In the control *WT* neurons, the signal for PSD-95 was indeed observed as dots along the dendrites (Fig. 4*C*). However, in the *MAGUIN*-deficient neurons, the signal for PSD-95 was decreased significantly (100.0 ± 6.1% in *WT* neurons and 70.8 ± 6.1% in *MAGUIN*-deficient neurons) (Fig. 4, *C* and *D*). Whereas there was no reduction in the PSD-95 cluster density in the *MAGUIN*-deficient neurons (number of clusters per 10 μm along dendrites: 8.0 ± 0.5 in *WT* neurons and 7.7 ± 0.4 in *MAGUIN*-deficient neurons) (Fig. 4, *C* and *E*). These results are consistent with the previous report using shRNA-mediated knockdown of *MAGUIN* in cultured hippocampal neurons (22) and are similar to the phenotypes observed in the *afadin*-deficient mossy fiber synapse (11). These results indicate that MAGUIN as well as afadin is required for the localization of PSD-95 at the synapses in cultured hippocampal neurons.

### Attenuation of postsynaptic response to glutamate and the cell surface localization of the AMPA receptor in MAGUIN-deficient cultured hippocampal neurons

**Figure 5:**
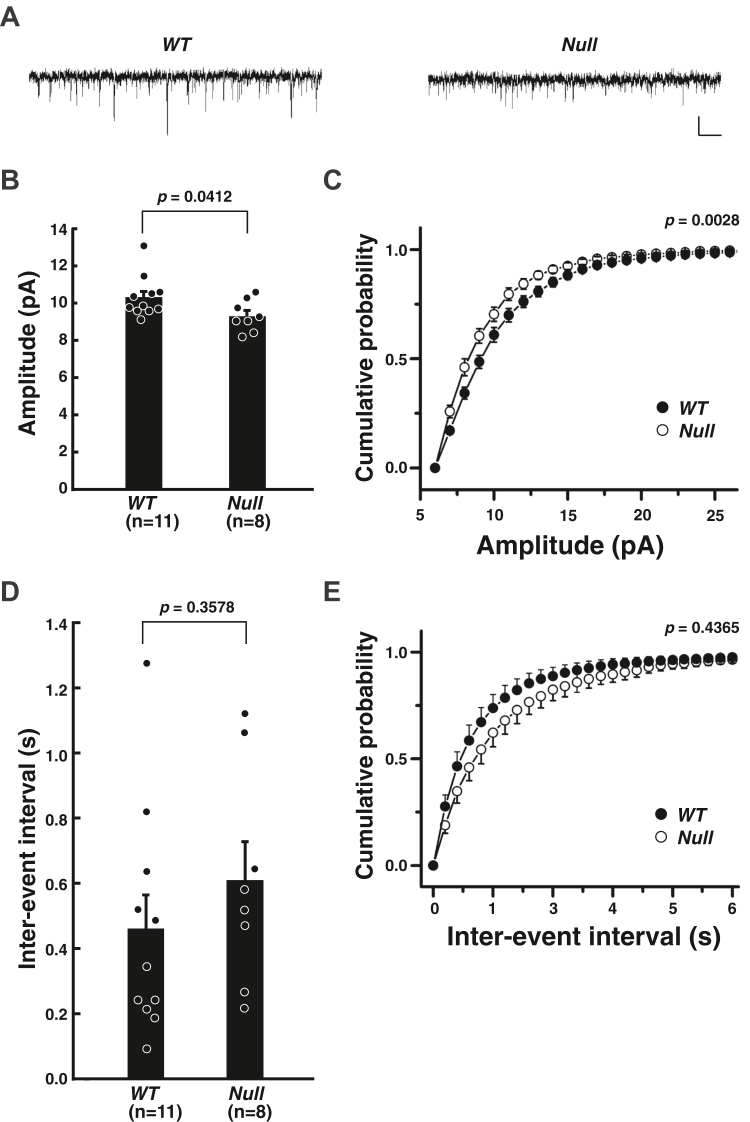
**Requirement of MAGUIN for the glutamatergic postsynaptic responsiveness incultured hippocampal neurons.***A*, examples of traces of mEPSCs recorded in the control *WT* and *MAGUIN*-deficient hippocampal neurons. *Vertical* and *horizontal* scale bars denote 10 pA and 500 ms, respectively. *B*, the average amplitudes of mEPSCs in the control *WT* and *MAGUIN*-deficient neurons. *C*, cumulative probabilities of amplitudes of mEPSCs in the control *WT* and *MAGUIN*-deficient neurons. *D*, the average inter-event intervals of mEPSCs in the control *WT* and *MAGUIN*-deficient neurons. *E*, cumulative probabilities of inter-event intervals of mEPSCs in the control *WT* and *MAGUIN*-deficient neurons. *WT* littermates were used as controls. mEPSC, miniature excitatory postsynaptic current; *Null*, *MAGUIN*-deficient. Statistical difference was analyzed by two-tailed Student’s *t* test in *B* and *D*, and Kolmogorov-Smirnov test in *C* and *E*. Error bars, SEM.

The properties of the postsynapses were next examined by electrophysiological recordings of cultured hippocampal neurons. Recordings were performed from typical pyramidal-shaped neurons identified based on the morphological features of the cells with flattened cell bodies and radially projecting dendrites. The amplitude of the miniature excitatory postsynaptic currents (mEPSC) was significantly smaller in the *MAGUIN*-deficient neurons, compared with that of the control *WT* neurons (10.4 ± 0.3 pA in *WT* neurons and 9.4 ± 0.3 pA in *MAGUIN*-deficient neurons), suggesting that the postsynaptic responsiveness to glutamate is reduced in the *MAGUIN*-deficient hippocampal neurons (Fig. 5, *A*–*C*). However, the inter-event interval of mEPSCs recorded in the cultured *MAGUIN*-deficient hippocampal neurons was not significantly different from that of the control *WT* hippocampal neurons (0.46 ± 0.10 s in *WT* neurons and 0.61 ± 0.12 s in *MAGUIN*-deficient neurons), suggesting that MAGUIN is not involved in the regulation of the number of glutamatergic excitatory synapses functioning in cultured hippocampal neurons (Fig. 5, *D* and *E*).

**Figure 6:**
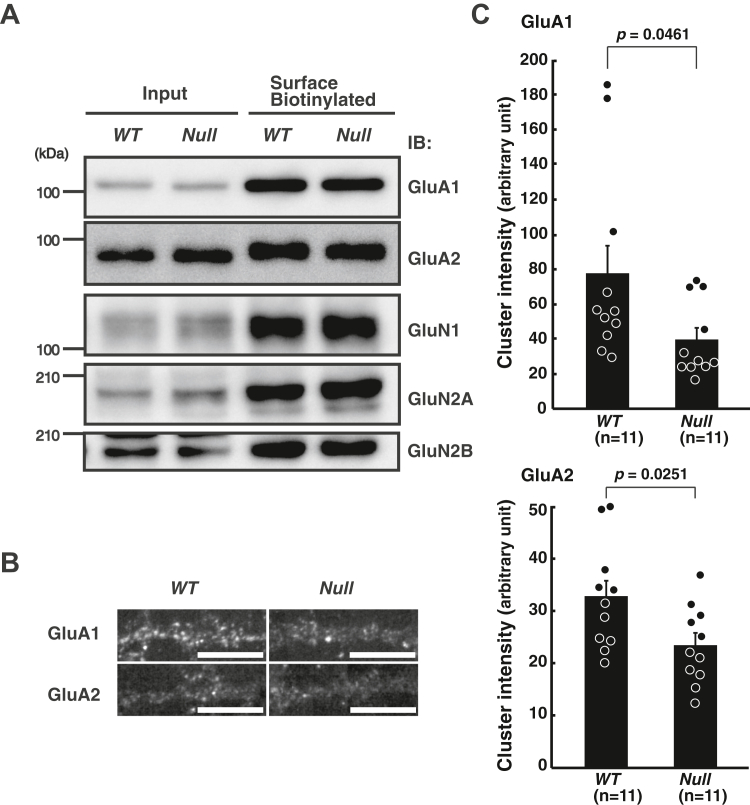
**Requirement of MAGUIN for the cell surface localization of the AMPA receptors incultured hippocampal neurons.***A*, the glutamate receptor subunits on the surface of the cultured hippocampal neurons obtained from control *WT* and *MAGUIN*-deficient mice. The cell surface proteins were labeled with membrane impermeable biotin, purified with streptavidin beads, and then were subjected to Western blotting along with lysates (input). *B* and *C*, reduced signal intensities of the AMPA receptor subunits on the surface of neurons. *B*, the AMPA receptor subunits, GluA1 and GluA2, on the surface of neurons, labeled with the Abs recognizing the extracellular regions of these AMPA receptor subunits. The scale bars represent 10 μm. *C*, the average signal intensity of the clusters of GluA1 (*upper graph*) or GluA2 (*lower graph*) on the cell surface of neurons. Each data point represents the mean signal intensity of 25 GluA1 or 25 GluA2 clusters per neuron. Eleven control and 11 *MAGUIN*-deficient neurons were measured for each analysis of the clusters of GluA1 or GluA2 in the primary hippocampal neuron cultures prepared three times. *WT* littermates were used as controls. *IB*, immunoblotting; *Null*, *MAGUIN*-deficient. Statistical difference was determined by two-tailed Student’s *t* test. Error bars, SEM. Ab, antibody; AMPA, α-amino-3-hydroxy-5-methyl-4-isoxazolepropionic.

Postsynaptic properties were further examined by the assessment of the expression of the AMPA-type ionotropic glutamate receptor subunit 1 (GluA1) and subunit 2 (GluA2) on the surface of the cultured hippocampal neurons, which mediate EPSCs and indirectly interact with PSD-95 to accumulate at synapses (23). The total amounts of surface expressions of GluA1 and GluA2, as well as N-methyl-D-aspartate (NMDA) receptor subunits, were not different between the control *WT* and *MAGUIN*-deficient neurons (Fig. 6*A*). The AMPA receptor subunit, GluA2, exists not only at the PSDs but also on the external surfaces of the neuronal somas, dendrites, and spines (24). Thus, even though the Western blot bands for the surface AMPA receptor subunits were not changed in the *MAGUIN*-deficient neurons, the accumulation of these proteins at synapses could be changed. Therefore, we examined the immunofluorescence signals of the GluA1 and GluA2 clusters on the cell surface and found that they were reduced by 49.0% and 28.4%, respectively, in the *MAGUIN*-deficient neurons, compared with those of the control *WT* neurons (Fig. 6, *B* and *C*). These observations are similar to the finding that the signal intensity of the GluA1 clusters on the cell surface is reduced in *afadin*-deficient hippocampal neurons (12). These results indicate that MAGUIN as well as afadin is involved in the postsynaptic functions in the hippocampal synapses by facilitating the cell surface localization of the AMPA receptor.

### No increase of the GABA_A_ receptor antagonist-induced seizure susceptibility by MAGUIN deficiency

**Figure 7:**
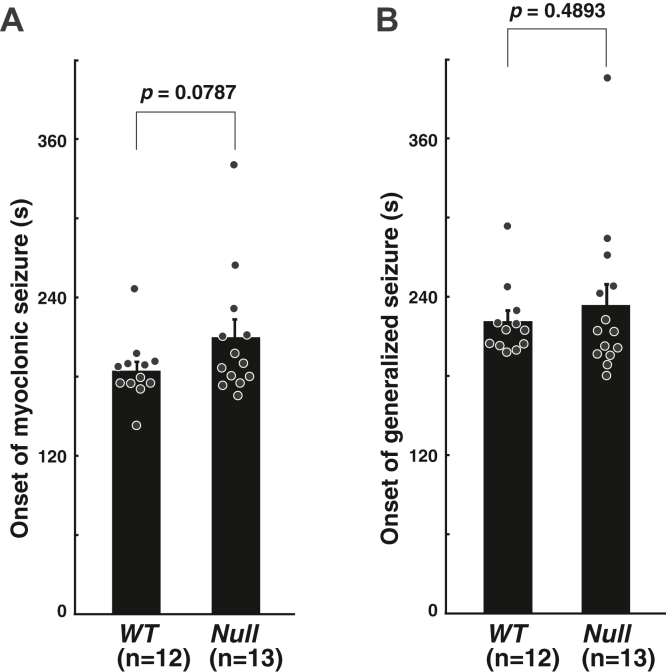
**No requirement of MAGUIN for the induction of epileptic seizures induced by exposure to flurothyl, a GABA**_**A**_**receptor antagonist, in mice.***A*, average latencies to the onset of myoclonic seizures. *B*, average latencies to the onset of generalized seizures. *WT* littermates were used as controls. *Null*, *MAGUIN*-deficient. Statistical difference was determined by two-tailed Student’s *t* test. Error bars, SEM.

Mutations in *MAGUIN/CNKSR2* were implicated in spontaneous seizure in both human patients and knockout mice (25). However, in the *MAGUIN*-deficient mice used in this study, no spontaneous seizures were observed under normal breeding conditions. Therefore, we examined whether a GABA_A_ receptor antagonist, flurothyl, would increase the seizure susceptibility in the *MAGUIN*-deficient mice. Test mice were placed in an airtight chamber filled with air containing volatile flurothyl. The latencies to myoclonic and generalized seizure were recognized as a reliable index of seizure threshold (26). The latency period of myoclonic seizure in the *MAGUIN*-deficient mice was rather slightly longer than in the control *WT* mice, although the difference was not statistically significant (185.5 ± 6.9 s and 211.0 ± 12.5 s in *WT* and *MAGUIN*-deficient, respectively) (Fig. 7*A*). The latency period of generalized seizure in the *MAGUIN*-deficient mice was almost the same as that in control *WT* mice (220.1 ± 7.7 s and 233.0 ± 15.7 s in *WT* and *MAGUIN*-deficient, respectively) (Fig. 7*B*). These results suggest that seizure susceptibility to flurothyl is not increased in the *MAGUIN*-deficient mice generated by us.

## Discussion

We previously showed that l-afadin, but not s-afadin, is involved in the formation of the PAJs of the mouse hippocampal mossy fiber synapse (13). In addition, it was previously shown that the longer isoform of AF-6 (synonym for l-afadin) regulates the spine morphogenesis through its PDZ and N-terminal RA domains (10). However, it remained unknown whether s-afadin is involved in the spine morphogenesis. In this study, we elucidated for the first time the role of s-afadin in the structural and functional differentiations of the PSDs of the mouse hippocampal synapses. We showed here that both l-afadin and s-afadin had the ability to form PSD-like structures in cultured hippocampal neurons and that s-afadin more preferentially bound to MAGUIN than l-afadin *in vivo* and *in vitro*, resulting in the formation of the PSDs.

The PSDs consist of many components, including PSD-95 and S-SCAM (27). The NMDA and AMPA glutamate receptors are anchored to the PSDs *via* binding to PSD-95 and S-SCAM (27). S-SCAM binds to not only PSD-95 and MAGUIN but also many other PSD components, such as SAPAP1, β-catenin, δ-catenin, and the NMDA glutamate receptor (14, 28, 29, 30, 31). Taken together with these previous studies, the present results demonstrate that s-afadin regulates the assembly of the PSD components through PSD-95 by binding to MAGUIN, potentially in cooperation with S-SCAM. The mechanism for the formation of the PSDs by l-afadin was not studied here but it is likely that l-afadin binds to a protein(s) different from MAGUIN and regulates the formation of the PSDs in a manner different from that of s-afadin. However, since l-afadin binds to MAGUIN-1 and -2, although to a lesser extent than s-afadin, a MAGUIN-dependent mechanism cannot be completely ruled out at present. Future analysis is required for this issue.

The precise mechanism for the more preferential binding of s-afadin to MAGUIN than l-afadin is not known but may be due to the structural difference between s-afadin and l-afadin. The only difference of the primary structure between them is that s-afadin lacks the third proline-rich domain and the FAB domain at the C-terminal region in l-afadin. We identified here that the MAGUIN-binding domain of s-afadin was the region containing two RA domains, which is located near its N terminus. Therefore, the intramolecular binding between the N-terminal RA domains and the FAB domain of l-afadin may hinder MAGUIN from binding to the RA domains of l-afadin. To elucidate the mechanisms for the different binding properties between l-afadin and s-afadin, three-dimensional structural analysis on them is essential.

We showed here that the PDZ domain of s-afadin is not required for the binding of MAGUIN to s-afadin, indicating that the binding of nectins to the PDZ domain of s-afadin is not involved in the formation of the PSDs by s-afadin in cooperation with MAGUIN. This present result is consistent with the previous finding that nectin-1 and nectin-3, which bind to the PDZ domain of l- and s-afadin, are not involved in the formation of the PSDs as well as the AZs by the analysis for the *nectin-1*-deficient mice and the *nectin-3*-deficient mice using electron microscopy (32).

We previously showed that l-afadin and s-afadin are mainly localized at the PAJs and partly at the SJs (8). The three-dimensional structure of the *afadin*-deficient hippocampal mossy fiber synapses showed that afadin was required for the AZ formation, assembly of the docked synaptic vesicles and synaptic vesicles in the readily releasable pool, and arborization of the postsynaptic spines (11). However, it remains unknown which splice variant of afadin regulates these processes. The present study showed that MAGUIN was involved in the postsynaptic functions of hippocampal synapses but not in the presynaptic ones by electrophysiological analysis. MAGUIN was shown to be localized in the synapses of cultured hippocampal neurons (14, 33). However, it is not clear whether MAGUIN is localized in the presynapses and/or postsynapses, because the Ab that is applicable to immunohistochemistry and immunoelectron microscopic analysis is not available currently. The present study indicates that MAGUIN is localized and functions, at least, in the postsynapses. On the other hand, we previously showed that afadin is involved in the NGL-3-LAR system-induced presynaptic differentiation of hippocampal neurons cooperatively with β-catenin and γ-catenin in a nectin-1-independent manner (8). It is unknown which isoform of afadin is involved in the NGL-3-dependent presynaptic differentiation. γ-Catenin more preferentially binds to s-afadin than l-afadin. Thus, s-afadin would play a more important role in the NGL-3-dependent presynaptic differentiation than l-afadin. However, further studies are needed to establish the roles of s-afadin in the presynapses.

We showed here that the postsynaptic responsiveness to glutamate and the surface expression of the AMPA receptor are reduced in the *MAGUIN*-deficient cultured hippocampal neurons. It was shown that PSD-95 and S-SCAM indirectly interact with the AMPA receptor through the transmembrane AMPA receptor regulatory proteins (23, 34). Therefore, it is possible that s-afadin regulates the localization of the AMPA receptor through MAGUIN in a PSD-95-dependent and/or a S-SCAM-dependent manner.

Other notable candidates functioning with the MAGUIN−afadin molecular complex are Traf2- and Nck-interacting protein kinase and Vilse/Arhgap39 (22, 35). Both bind to MAGUIN and regulate PSD morphogenesis in cooperation with small G proteins. Afadin is a critical regulator of several small G proteins, including Rap and Rac (36). By exploring these molecular linkages consisting of s-afadin, MAGUIN, Traf2- and Nck-interacting protein kinase, and/or Vilse, the mechanisms of postsynapse formation will be understood in more detail.

MAGUIN was identified to be a mammalian homologue of the *Drosophila Cnk* gene product, which regulates the Ras-Raf-MAPK signaling (15). MAGUIN was further shown to bind to Raf-1 (16). In this current study, Raf-1 was not examined in the *MAGUIN*-deficient hippocampal neurons. The Ras-Raf-MAPK signaling is involved in the synaptic plasticity in the hippocampus and regulates the gene expression of proteins including the PSD proteins (37). Thus, the possible roles of MAGUIN in the Ras-Raf-MAPK signaling need to be clarified. MAGUIN was further shown to be required for the dendritic spine formation (35). The structural plasticity of dendritic spines is related to the synaptic efficacy and learning and memory (38). Therefore, s-afadin might be involved in the synaptic plasticity through the binding to MAGUIN.

MAGUIN is reported to have association with some neuropsychiatric diseases. Partial deletion and deficiency of *MAGUIN*/*CNKSR2* and point mutations in *MAGUIN*/*CNKSR2* are involved in nonsyndromic X-linked intellectual disability accompanied with epilepsy and aphasia (17, 18, 20, 39) and *MAGUIN*/*CNKSR2* is associated with schizophrenia (40). Moreover, the expression of afadin is reduced in the anterior cingulate cortex of schizophrenia patients (41). Therefore, if the roles of MAGUIN and afadin in the development of the brain and the maintenance of synapses and neural circuits as well as their mechanisms of action were made clear, pathogenesis of these diseases would be better understood.

In this study, apparent epileptic seizures and facilitation of flurothyl-induced seizures were not observed in the *MAGUIN*-deficient mice established here. Therefore, epilepsy in the human patients with mutations in *MAGUIN/CNKSR2* may not be simply caused by the defects in the excitatory synaptic transmission. In contrast, it was recently reported that *Cnksr2*-deficient mice of a different strain from the one used in this study display spontaneous seizure, and net increase of cortical neuronal activity, as well as several behavioral abnormalities (25), although the targeting strategies of these two strains, in which exon 2 was deleted, were quite similar. The clear difference is the genetic background of the mouse strains: C57BL/6J was used by Erata and colleagues, while C57BL/6N was used in this study. Importantly, C57BL/6J and C57BL/6N are known to show different flurothyl-induced seizure susceptibilities (42). The detailed mechanisms of the difference need to be clarified in the future.

## Experimental procedures

### Antibodies

A rabbit l/s-afadin pAb recognizing both of l-afadin and s-afadin was prepared as described (4). A rabbit antiserum was raised against a synthetic peptide corresponding to aa 759-777 (RKTASQRRSWQDLIETPLT) of mouse MAGUIN (NP_808419). The antiserum was affinity-purified with the antigen and the purified antiserum was used as a MAGUIN pAb. The Abs listed below were purchased from commercial sources: rabbit GFP pAb (Thermo Fisher Scientific); mouse V5 mAb (Thermo Fisher Scientific); mouse actin mAb (BD Transduction Laboratories); mouse PSD-95 mAb (clone K28/43) (NeuroMAB); mouse GluA1 mAb (Merck); mouse GluA2 mAb (Merck); and chicken MAP2 pAb (abcam), mouse GluN1 mAb (BD Transduction Laboratories), rabbit GluN2A pAb (Frontier Institute), and rabbit GluN2B pAb (Frontier Institute). Alexa Fluor-conjugated goat or donkey secondary Abs (Thermo Fisher Scientific and Jackson ImmunoResearch) were used for immunocytochemistry.

### Constructs

The mammalian expression plasmids for EGFP-tagged rat l-afadin, EGFP-tagged rat s-afadin, and bacterial expression plasmid for GST-rat afadin NN (aa 2–500) were described (21). The complementary DNA coding mouse *MAGUIN-1* (clone ID: 6405389) was obtained from the I.M.A.G.E. consortium. The expression plasmid for an EGFP-tagged s-afadin mutant lacking the RA domains (aa 350–1663), V5-tagged plasmids for full-length MAGUIN-1, full-length MAGUIN-2, MAGUIN-2-N (aa 1–572), and MAGUIN-2-C (aa 567–896) were constructed using PCR-based standard molecular biology techniques.

### Immunoprecipitation

All the procedures were conducted at 4 °C or on ice. For the analysis of endogenous proteins, the brains of male ICR mice at postnatal day 21 were homogenized in 8-fold brain volumes of a buffer containing 20 mM Hepes (pH 7.4), 320 mM sucrose, 5 mM EDTA, and 5 mM EGTA supplemented with protease inhibitors: 1 mM phenylmethylsulfonyl fluoride, 10 μg/ml aprotinin, 10 μg/ml leupeptin, 1 μg/ml pepstatin, and 500 μM benzamidine. The homogenate was subjected to centrifugation at 3,000*g* for 15 min. The supernatant was further subjected to centrifugation at 38,400*g* for 15 min. The pellet was washed with wash buffer containing 20 mM Hepes (pH 7.4), 5 mM EDTA, and 5 mM EGTA supplemented with the protease inhibitors, and then resuspended in 0.25% dodecyl maltoside (DDM, Dojindo) in buffer A containing 100 mM NaCl, 20 mM Hepes (pH 7.4), 5 mM EDTA, and 5 mM EGTA supplemented with the protease inhibitors. After a 3-h incubation on a rocking platform, insoluble aggregates were removed by centrifugation at 40,000*g* for 30 min and the supernatant (light membrane fraction) was obtained. The supernatant was then incubated on a rocking platform with the control rabbit IgG (Jackson ImmunoResearch Laboratories), the l-afadin pAb or the l/s-afadin pAb overnight. Each of the Abs was covalently coupled to protein A magnetic beads (New England Biolabs). After extensive washes, proteins captured by the beads were eluted with a buffer containing 0.1 M glycine (pH 2.5) and 0.25% DDM. The samples were neutralized with 100 mM Tris–HCl (pH 8.8) and boiled in an SDS-sample buffer and then were subjected to SDS-PAGE followed by Western blotting. For the analysis of recombinant proteins, HEK293 cells were transfected with the indicated plasmids using Lipofectamine 3000 (Thermo Fisher Scientific) or calcium phosphate and were cultured for 2 days. The transfected cells were washed with ice-cold PBS and were collected by centrifugation at 8,000*g* for 2 min. The cell pellets were resuspended in 0.25% DDM in buffer A. After a 3-h incubation on a rocking platform, insoluble aggregates were removed by centrifugation at 40,000*g* for 30 min and the supernatants were obtained. The supernatants were incubated with the EGFP pAb on a rocking platform overnight followed by 2-h incubation with protein-G Sepharose beads. After extensive washes with 0.25% DDM in buffer A, the beads were boiled in the SDS-sample buffer and then the bound proteins were subjected to SDS-PAGE followed by Western blotting.

### GST pulldown assay

GST and GST-fused afadin-NN were expressed in *Escherichia coli* and the cells were sonicated in a sonication buffer containing 50 mM Tris–HCl (pH 7.5), 150 mM NaCl, 0.1% Triton X-100, 1 mM DTT, 1 mM phenylmethylsulfonyl fluoride, and 10 μg/ml leupeptin. The homogenates were clarified by centrifugation at 20,380*g* for 15 min and the supernatants were obtained. The supernatants were then incubated with glutathione-Sepharose 4B (GE Healthcare) at 4 °C for 1 h to immobilize GST-fused proteins. After being washed with 0.25% DDM in buffer A, the beads were incubated with the cell lysates prepared from HEK293 cells exogenously expressing V5-MAGUIN-2 in the same buffer. After a 3-h incubation, the beads were extensively washed with the 0.25% DDM in buffer A and bound proteins were subjected to SDS-PAGE, followed by Western blotting with a V5 mAb. The membranes were stained with Amido Black 10B (Bio-Rad Laboratories) to confirm protein loading.

### Western blotting

The lysates of the light membrane fraction of mouse brain and the immunoprecipitated samples were mixed with the SDS sample buffer and boiled for 5 min. The samples were then separated by SDS-PAGE and transferred to polyvinylidene difluoride membranes (Merck). After being blocked with 5% skim milk in Tris buffered saline (TBS), 20 mM Tris–HCl (pH 7.4) and 150 mM NaCl with 0.05% Tween 20 (TBS-T), the membranes were incubated with the indicated Abs. After being washed with TBS-T three times, the membranes were incubated with the horseradish peroxidase-conjugated rabbit or mouse IgG pAb (Jackson ImmunoResearch Laboratories). The signals for the proteins were detected using Immobilon Western Chemiluminescent horseradish peroxidase Substrate (Merck).

### Mice

The *afadin*-floxed mice and *nestin*-cre mice were described (43, 44). They were kept on a C57BL/6J background. The heterozygous mice carrying the *afadin* conditional allele are referred to as *Afadin*^+/f^. The mutant and control *WT* samples were prepared from the same litters. A *MAGUIN/Cnksr2* null mouse line was generated in this study by using a targeting vector (PRPGS00027_A_G05) obtained from the *trans*-NIH Knockout Mouse Program (45). After electrotransformation of the targeting vector, C57BL/6N mouse blastocyst-derived RENKA embryonic stem cells (46) harboring the targeted allele were selected for G418 resistance. Right clones were injected into ICR 8-cell stage embryos that were then introduced into pseudopregnant females to yield chimeric mice. The chimeric mice were bred with C57BL/6N mice to generate heterozygous mice carrying the mutant allele. Subsequently, the heterozygous mice were mated with C57BL/6N mice expressing FlpE recombinase in the germ cell lineage to excise the lacZ and Neo cassette flanked by flippase recognition target sites. The heterozygous mice with the targeted allele after FlpE-mediated recombination were further mated with C57BL/6N mice expressing Cre recombinase in the germ cell lineage to excise the exon 2 flanked by *lox*P sites. The mutant allele finally generated in this way is referred to as *MAGUIN*^+/−^. Mice carrying the mutant allele were genotyped by PCR using a primer set: F1, 5′-GATTGAACCTAGCACAGTCTGTAGCCT-3′; R1, 5′-GCTACCCCTACTTTCAGAGTTATGTACATCA-3′; and R2, 5′-CACACTGTGATACACTACAGCTTCCACAA-3′. The PCR reaction consists of 2 min at 95 °C followed by 30 cycles of 30 s at 95 °C, 30 s at 62.5 °C, and 90 s at 72 °C. The primer pair, F1 and R1, gives a 470-bp band for a *WT* allele and the primer pair, F1 and R2, gives a 607-bp band for a mutant allele and a faint 1260-bp band for a *WT* allele. The *MAGUIN*^+/−^ mice used in the present study have been crossed with pure C57BL/6N mice more than ten times. All animal experiments were performed in accordance with the guidelines of the institution and approved by the administrative panel on laboratory animal care of Kobe University and Kitasato University. This study was approved by the president of Kobe University after being reviewed by the Kobe University Animal Care and Use Committee (Permit Numbers: P130205 and 2-24-03-02) and by the president of Kitasato University after being reviewed by the Kitasato University Animal Care and Use Committee (Permit Number: 2020-150).

### Dissociated culture of hippocampal neurons and reexpression of EGFP-afadin in afadin-deficient hippocampal neurons

Dissociated hippocampal neurons were prepared as described (47). In brief, the hippocampal neurons dissociated with trypsin were plated at a density of 5.0 to 7.0 × 10^3^ cells/cm^2^ on poly-L-lysine-coated coverslips in Neurobasal medium (Thermo Fisher Scientific) containing B-27 supplement (Thermo Fisher Scientific) and GlutaMAX (Thermo Fisher Scientific) and cultured in a 5% CO_2_ incubator. For the reexpression of EGFP-l-afadin or EGFP-s-afadin in the *afadin*-deficient hippocampal neurons prepared from *Afdn*^f/f^;*nestin*-cre mice, pEGFP, pEGFP-l-afadin, pEGFP-s-afadin, or pEGFP-s-afadinΔRA was introduced with an Amaxa Nucleofector (Lonza) at the time of seeding according to the manufacturer’s protocol. At 14 days *in vitro*, the cells were fixed and were immunostained with the indicated Abs.

For the measurement of the signal intensities of the PSD-95 clusters and the cell surface AMPA receptor subunit clusters and the electrophysiology in cultured *MAGUIN*-deficient neurons, dissociated hippocampal neurons were prepared as described (47). In brief, the hippocampal neurons dissociated with trypsin were plated at a density of 5.0 to 7.0 × 10^3^ cells/cm^2^ on poly-L-lysine-coated coverslips in minimal essential medium containing 10% fetal calf serum overnight, followed by medium switching with Neurobasal medium containing B-27 supplement and GlutaMAX on the next day, and cultured in a 5% CO_2_ incubator at 37 °C. The cells were subjected to the experiments at 18 to 24 days *in vitro*.

### Detection of the cell surface AMPA receptor

Neurons were washed twice with ice-cold PBS and incubated with a membrane impermeable biotinylation reagent, Sulfo-NHS–SS–biotin (Thermo Fisher Scientific; 0.25 mg/ml in PBS), for 15 min on ice. Neurons were then washed twice with PBS containing 50 mM NH_4_Cl and twice with PBS before being scraped into ice-cold lysis buffer (150 mM NaCl, 20 mM Hepes (pH 7.4), 1% Triton-X-100, 0.1% SDS, 2 mM EDTA, and protease inhibitors). After a 2-h incubation on a rocking platform, insoluble aggregates were removed by centrifugation at 40,000*g* for 30 min and the supernatants were obtained and used as inputs. The inputs were incubated on a rocking platform with streptavidin conjugated Sepharose beads for 2 h. After extensive washes with the buffer, the beads were boiled in the SDS-sample buffer and the supernatants were used as samples. Inputs and samples were subjected to SDS-PAGE followed by Western blotting.

### Immunocytochemical analysis

For immunostaining of cultured hippocampal neurons, the cells were fixed with a fixative containing 2% paraformaldehyde, 4% sucrose, 1 mM sodium pyruvate, Hanks’ balanced salt solution containing 1 mM CaCl_2_ and 1 mM MgCl_2_ (Thermo Fisher Scientific) in 10 mM Hepes (pH 7.4) at 37 °C for 10 min. The fixed cells were permeabilized with 0.25% Triton-X in TBS containing 1 mM CaCl_2_ and 0.05% Tween-20 (T-TBS) at room temperature for 5 min and then blocked in T-TBS containing 10% goat serum at room temperature for 20 min. Then, the cells were incubated with primary Abs in T-TBS containing 10% goat serum at 4 °C overnight. After three washes in T-TBS at room temperature for 5 min each, the cells were incubated with Alexa Fluor-conjugated secondary Abs (Thermo Fisher Scientific) at room temperature for 45 min, followed by three washes for 5 min each with T-TBS. The samples were then mounted in a FluorSave reagent (Merck). To examine the effects of EGFP-afadin reexpression on the localization of PSD-95 in the *afadin*-deficient hippocampal neuron, the fields, which contained EGFP-positive and EGFP-negative neuronal dendrites (judged by the presence of MAP2 immunoreactivities) of the neurons showing typical pyramidal cell-like morphologies, were chosen and imaged with an LSM510 META confocal laser scanning microscope (Carl Zeiss) using identical image acquiring setting throughout the experiments without saturation of the signals. Maximum signal intensity projection images were created from around ten confocal images collected at a 0.3 to 0.4-μm step along the z-axis.

For the analysis of the reexpressions of EGFP-afadin variants in the *afadin*-deficient hippocampal neurons, the signal intensities of the PSD-95 clusters were measured using the ImageJ software (https://imagej.net) and averaged for the transfected and untransfected neurons in the same field of view and the ratio of them was calculated per each image. For the measurement of the signal intensity of the PSD-95 clusters in *MAGUIN*-deficient neurons, cells were fixed, permeabilized, and then stained with the PSD-95 mAb. For the measurement of the signal intensity of the AMPA receptor subunit clusters on the cell surface of hippocampal neurons, cells were labeled alive using the GluA1 mAb or GluA2 mAb without detergents. The neurons were then fixed and incubated with fluorescent secondary Abs and imaged with an LSM710 confocal laser scanning microscope (Carl Zeiss). In detail, for the measurement of the signal intensity of the PSD-95, GluA1, or GluA2 clusters, the total signal intensity in a circle containing a single PSD-95, GluA1, or GluA2 cluster was measured, and the total signal intensity in a circle of the same diameter in the vicinity of the clusters, which did not contain the PSD-95, GluA1, or GluA2 clusters, was measured to obtain the background signal. Then, the latter value was subtracted from the former to obtain the net value of signal intensity. The mean signal intensity of 50 PSD-95 clusters, 25 GluA1 clusters, or 25 GluA2 clusters per neuron was calculated and represented as each data point in the graphs.

### Electrophysiology

Whole-cell patch-clamp recordings from cultured hippocampal neurons were performed as described (48, 49, 50). The extracellular solution contained (in mM) 119 NaCl, 2.5 KCl, 25 Hepes (pH 7.4), 30 D-glucose, 2 CaCl_2_, and 2 MgCl_2_. The mEPSCs were recorded in the extracellular solution contained 1 μM tetrodotoxin (FUJIFILM Wako Pure Chemical), 10 μM gabazine (Toronto Research Chemicals), and 1 μM strychinine (Merck). Currents were recorded at room temperature under voltage-clamp (−70 mV) conditions. For measurements of mEPSCs, electrodes were filled with solution containing (in mM), 125 K-methanesulfonate, 6 KCl, 2 MgCl_2_, 10 Hepes (pH 7.4), 0.6 EGTA, 3.2 Mg-ATP, and 1.2 Na-GTP, and membrane potentials were corrected for the liquid junction potential (9 mV). Recordings were performed with EPC 10 amplifier (HEKA Elektonik GmbH). Records were filtered at 1 to 2 kHz and acquired at 10 kHz. Series resistance values were <20 MΩ. mEPSCs were analyzed using Clampfit 10.2 (Molecular Devices), Origin 2015 (OriginLab) and Minianalysis program 6.0.3 (Synaptosoft). Recordings were performed on typical pyramidal-shaped neurons as described (48, 51). In detail, these cells were identified from the other neurons, including inhibitory neurons, based on their morphological features of the cells with flat cell bodies and radially projecting dendrites. In addition, under the experimental conditions similar to those in this study, CNQX, an inhibitor of AMPA-/kainate-type glutamate receptors, is shown to abolish evoked EPSCs at −70 mV in cultured hippocampal neurons (48).

### Examination of susceptibility to flurothyl-induced seizure

The experiments were conducted in accordance with the animal experiment protocol as described (26). The experiments were conducted in a validated chemical fume hood to avoid any flurothyl leak. Mice were individually placed in an airtight chamber: a 2-l glass beaker with an order-made O-ring sealed cap and a sealed plastic cup as the room to evaporate flurothyl (bis(2,2,2-trifluoroethyl) ether) (Merck). The bottom of the plastic cup had multiple holes through which evaporated flurothyl reached to mice. Mice were allowed to habituate to the chamber for 1 min prior to the administration of 10% flurothyl dissolved in 95% ethanol that were infused through a 5-ml syringe onto gauze pads suspended in the plastic cup at a rate of 0.2 ml/min. Seizure behaviors were recorded with a video camera and the following events were scored in a genotype-blinded way: (1) latency to the first myoclonic jerk (*i.e.*, brief, but severe, contractions of the neck and body musculature occurring while the mouse maintains postural control); and (2) latency to the first generalized seizure (*i.e.*, convulsions resulting in a loss of postural control). Upon observation of a generalized seizure, mice were immediately removed from the chamber, exposed to a fresh air to facilitate cessation of the seizure. The chamber was cleaned with 70% ethanol, well dried up, and the gauze pads were replaced to new ones between trials.

### Statistical analysis

Statistical analysis of the difference in means between two groups was performed with the two-tailed Student’s *t* test or two-tailed paired *t* test. For the analysis of the difference in means among three groups, one-way ANOVA and post hoc Scheffe tests were performed. The criterion for statistical significance was set at *p* < 0.05. All values are reported as mean ± SEM.

### Southern blotting

Southern blotting was performed with a standard method using a *Neo* probe.

## Data availability

All data are included within this article.

## Conflict of interest

The authors declare that they have no conflicts of interest with the contents of this article.
